# Up-Regulation of Endothelin Receptors Induced by Cigarette Smoke — Involvement of MAPK in Vascular and Airway Hyper-Reactivity

**DOI:** 10.1100/tsw.2010.204

**Published:** 2010-11-04

**Authors:** Yaping Zhang, Lars Edvinsson, Cang-Bao Xu

**Affiliations:** ^1^ Division of Experimental Vascular Research, Institute of Clinical Science in Lund, Lund University, Sweden; ^2^ Division of Ear, Nose and Throat Diseases, Department of CLINTEC, Karolinska Institutet, Karolinska University Hospital/Huddinge, Sweden

**Keywords:** cigarette smoke, vasculature, airway, hyper-reactivity, endothelin receptor, MAPK

## Abstract

Cigarette smoke exposure is well known to cause cardiovascular and airway diseases, both of which are leading causes of death and disability in the world. However, the molecular mechanisms that link cigarette smoke to cardiovascular and airway diseases are not fully understood. Vascular and airway hyper-reactivity plays an important role in the pathogenesis of cardiovascular and airway diseases. Recent studies have demonstrated that endothelin receptor up-regulation mediates vascular and airway hyper-reactivity in response to endothelin-1 (ET-1, endothelin receptor agonist) in cardiovascular and airway diseases. In the vasculature and airways, the main functional consequences of up-regulated endothelin receptors by cigarette smoke exposure are enhanced contraction and proliferation of the smooth muscle cells, which subsequently result in abnormal contraction (spasm) and adverse proliferation (remodeling) of the vasculature and airways. The structural alteration by adverse remodeling involves changes in cell growth, cell death, cell migration, and production or degradation of the extracellular matrix. This review focuses on cigarette smoke exposure that induces activation of intracellular mitogen-activated protein kinase (MAPK) and subsequently results in the up-regulation of endothelin receptors in the vasculature and airways, which mediates vascular and airway hyper-reactivity, one of the important pathogenic characteristics of cardiovascular and airway diseases. Understanding the molecular mechanisms of how cigarette smoke causes up-regulation of endothelin receptors in the vasculature and airways may provide new strategies for the treatment of cigarette smoke—associated cardiovascular and lung diseases.

## INTRODUCTION

Cardiovascular and respiratory diseases are leading causes of death and disability worldwide. Both active and passive exposure to cigarette smoke are strongly associated with cardiovascular diseases such as atherosclerosis, coronary heart disease, stroke, myocardial infarction, aortic aneurysm, and peripheral vascular diseases[1,2]. In airways, cigarette smoke exposure induces chronic obstructive pulmonary disease (COPD) and chronic bronchitis, exacerbates asthma, and is also associated with lung cancer[3,4]. Although the clinical outcomes of cardiovascular and airway diseases have been improved, new pharmacological tools that target the molecular mechanisms responsible for developing cardiovascular and airway diseases still need to be elucidated.

Endothelin-1 (ET-1) is the most potent endogenous vasoconstrictor[5,6] and bronchoconstrictor[7,8], as well as a strong growth factor[9]. The circulating levels of ET-1 are elevated after exposure to cigarette smoke in humans[10] and in rodents[11], suggesting that ET-1 is involved in cigarette smoke–induced vascular and airway hyper-reactivity. However, there is limited knowledge about the underlying molecular mechanisms of how cigarette smoke exposure leads to vascular and airway hyper-reactivity in the process of cardiovascular and airway diseases. We and others have studied the effects of cigarette smoke exposure on endothelin receptor expression in the vasculature and airways, and how this is associated with vascular and airway hyper-reactivity. The results show that up-regulation of endothelin receptors is one of the mechanisms associated with the development of vascular and airway hyper-reactivity. Furthermore, cigarette smoke exposure results in activation of mitogen-activated protein kinase (MAPK), and increases expression and function of endothelin receptors. Thus, targeting MAPK-mediated up-regulation of endothelin receptors might be a novel and promising therapeutic target for treating vascular and airway hyper-reactivity, and cardiovascular and airway diseases.

## ENDOTHELIN-1 AND ENDOTHELIN RECEPTORS

ET-1 was initially isolated and identified from conditioned medium of cultured porcine endothelial cells, and was shown to be a potent vasoconstrictor peptide consisting of 21 amino acids[5]. It exerts an extremely diverse set of actions that influence homeostatic mechanisms throughout the body[12]. Big ET-1 (38 amino acids) is the precursor for ET-1, and it is derived from preproET-1 (212 amino acids) and further cleaved by endothelin-converting enzyme (ECE) into ET-1[13] (Fig. 1). Endothelial cells are a major source of ET-1, making this peptide fairly ubiquitous, and the constitutive release of ET-1 from the endothelium may contribute to the basal vascular tone[14]. Under proinflammatory conditions, vascular and airway smooth muscle cells (SMCs) and pulmonary epithelial cells also produce ET-1[15].

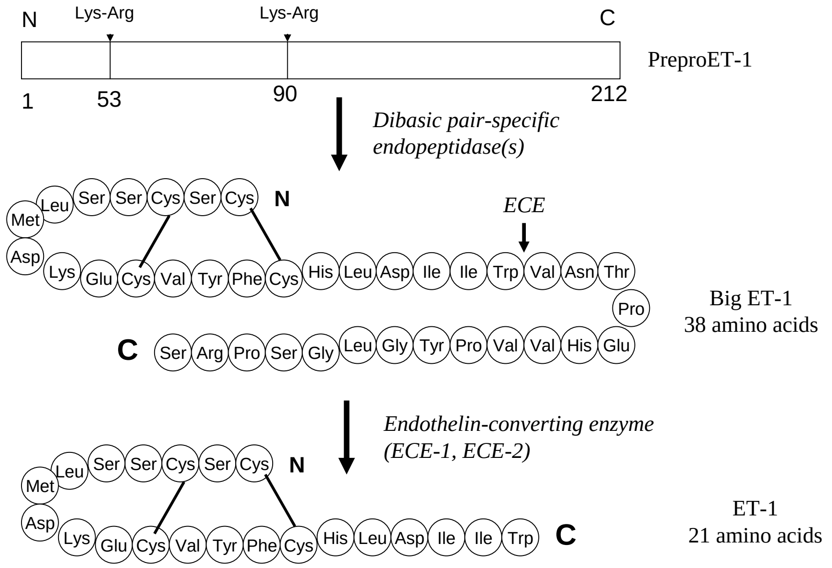

**FIGURE 1.** The biosynthesis and processing of ET-1.

Two major endothelin receptors, endothelin type A ($ET_A$) and type B ($ET_B$), are recognized. The $ET_B$ receptors are further divided into two subtypes, $ET_{B1}$ and $ET_{B2}$[16]. Activation of $ET_A$ and $ET_{B2}$ receptors induce the SMC contraction, proliferation, and migration. However, $ET_{B1}$ receptors that are located on the endothelium mediate vasodilatation[16,17]. In cardiovascular disease, this endothelium-dependent relaxation is often absent due to endothelium dysfunction induced by risk factors such as cigarette smoke exposure[18]. Up-regulation of $ET_A$ and $ET_{B2}$ receptors and loss of $ET_{B1}$ receptor function may mediate vascular and airway hyper-reactivity in response to ET-1 in the vasculature[9,19] and airways[20,21].

In the human cardiovascular system, ET-1 is the most important isoform of the peptides that belong to the endothelin family, as it induces a long-lasting potent vasoconstriction[5,22] and stimulates proliferation of vascular SMCs[9,19]. ET-1 binds tightly to the $ET_A$ receptor and gives rise to a strong, long-lasting contraction with a low receptor turnover[23]. This prolonged effect may be due to the localization of the receptors in caveolae with a low rate of internalization[24]. The $ET_B$ receptor, on the other hand, is rapidly internalized and inactivated through phosphorylation following activation[25] and, thus, evokes a more transient response. The intracellular fate after activation differs between $ET_A$ and $ET_B$ receptors; the former is directed to the pericentriolar recycling compartment and reappears at the plasma membrane, whereas the latter is directed to lysosomes for degradation[26] (Fig. 2). In addition, it has been shown that $ET_B$ receptors play an important role in the clearance of ET-1[27].

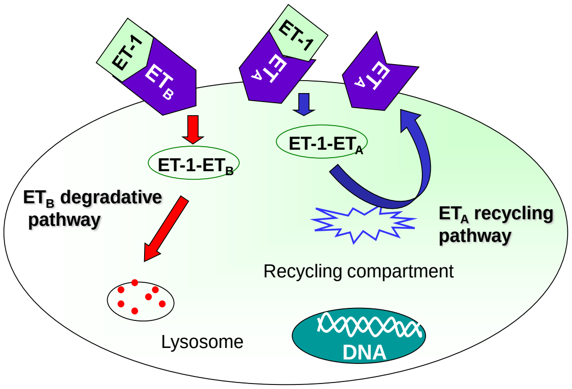

**FIGURE 2.** Schematic diagram of intracellular fates of $ET_A$ and $ET_B$ receptors. The $ET_A$ receptors are directed to the pericentriolar recycling compartment and reappear at the plasma membrane, whereas $ET_B$ receptors are directed to lysosomes for degradation.

In airways, the $ET_B$ receptor predominates on the surface of human bronchial SMCs by about 82–88% of the total endothelin receptor population[28]. In murine airway SMCs, $ET_A$ and $ET_B$ receptors are present in more-or-less equal proportions[29]. Activation of the $ET_{B2}$ receptors causes airway contraction, whereas stimulation of the $ET_A$ receptors has been associated with effects such as mediator release and recruitment of inflammatory cells, as well as contraction[30]. Both $ET_A$ and $ET_B$ receptors are present on the airway epithelium as well, where they can induce relaxation through the release of nitric oxide[31].

## UP-REGULATION OF ENDOTHELIN RECEPTORS

### Vasculature

Elevated plasma levels of ET-1 are seen after exposure to cigarette smoke in humans[10] and in rats[11]. Rats exposed to cigarette smoke exhibit vascular hyper-reactivity, which is significantly attenuated by administration of bosentan ($ET_A$ and $ET_B$ receptor antagonist)[32], suggesting that ET-1 release and endothelin receptor activation are involved in smoke-induced vascular hyper-reactivity. Our studies have demonstrated that dimethyl sulfoxide (DMSO)–soluble (lipid-soluble) particles from cigarette smoke (DSPs), at a concentration equivalent to the plasma level seen in smokers, not only reduce the vasodilator function of vascular endothelial cells[18], but also induce up-regulation of $ET_A$[33] and $ET_B$[34] receptor–mediated contractions in resistance arteries of rats. Recently, we compared the effects of cigarette smoke particles extracted by heptane (heptane-soluble smoke particles, HSPs), by water (water-soluble smoke particles, WSPs), and by DMSO (DMSO-soluble smoke particles, DSPs), which represent lipophilic, hydrophilic, and ambiphoteric constituents from the cigarette smoke, respectively. The results reveal that HSPs and DSPs, but not WSPs, increase the contractile responses to the $ET_B$ receptor agonist in rat mesenteric arteries[19]. In addition, the cigarette smoke extract DSPs increase ET-1–induced human aortic SMC proliferation, while blockage of nicotinic receptors had no effects, suggesting that nicotine receptors are not involved in such effects[19]. Up-regulation of $ET_B$ receptors has been observed in human blood vessels of patients with ischemic heart disease[35], after experimental ischemic stroke in rats[36], in human and murine hypertension[22,37,38], as well as in atherosclerotic plaques and neointimas[39]. The data suggest that the up-regulation of endothelin receptors is an important molecular mechanism that could play an essential part in the development of cardiovascular diseases associated with cigarette smoke.

## Airways

Passive cigarette smoke exposure results in airway hyper-responsiveness[40,41] and increases the bronchial hyper-responsiveness in allergic airway disease[42,43]. Chronic passive exposure to tobacco smoke increases airway reactivity to bradykinin and/or ET-1 in guinea pigs[44] and in mice[21]. Rat bronchial segments exposed to cigarette smoke extract DSPs exhibit increased expression of airway SMC $ET_A$ and $ET_B$ receptors with enhanced contractile responses[45]. In addition, cigarette smoke exposure rapidly causes proliferation of intrinsic cells in the airways and small vessels; this effect may eventually lead to airway wall muscular hyperplasia and fibrosis (small airways disease), and to vascular changes associated with pulmonary hypertension[46]. The $ET_A$ receptor antagonist BQ-610 blocks cigarette smoke–induced mitogenesis in rat airways and vessels[47], suggesting that cigarette smoke–induced cell proliferation of the airways and pulmonary arterial vessels is, at least in part, mediated through activation of the $ET_A$ receptors. In allergy mice, $ET_A$ and $ET_B$ receptor antagonists SB-217242 and bosentan show inhibitory effects on airway hyper-responsiveness[48,] and decrease the release of TNF-α and IL-1β in bronchial lavage fluids in eosinophilic airway inflammation[30]. Thus, endothelin receptor activation is involved in airway hyper-reactivity associated with cigarette smoke exposure.

Cigarette smoke exposure is well-known to associate with COPD and its complication, pulmonary hypertension. The pulmonary vasculature in patients with pulmonary hypertension in COPD is markedly abnormal, and shows increased intimal and medial thickening that cause luminal narrowing and vascular obstruction of the small pulmonary arteries. These vascular changes lead to an increase in pulmonary vascular resistance and elevation of pulmonary artery pressures. The traditional view is that pulmonary hypertension is secondary to loss of the vascular bed from emphysema. However, studies have demonstrated that in a guinea pig model of chronic cigarette smoke exposure, although some of these animals developed pulmonary hypertension, there was no loss of the capillary vascular bed[49]. Similarly, the National Emphysema Treatment Trial failed to find any correlation between mean pulmonary artery pressure and emphysema[50]. The association between pulmonary hypertension and lung disease is well recognized, but the underlying molecular mechanisms are unclear. Based on our *in vivo*[21] and *in vitro*[19] findings, we believe that cigarette smoke–induced damage of pulmonary vasculatures and airways occurs simultaneously.

## MAPK-MEDIATED UP-REGULATION OF ENDOTHELIN RECEPTORS

The molecular mechanisms responsible for the cigarette smoke–induced up-regulation of endothelin receptors, and the subsequent vascular and airway hyper-reactivity, are poorly understood. Recent studies suggest that intracellular MAPK signaling may play a key role in mediating endothelin receptor up-regulation. The intracellular MAPK signaling consists of extracellular signal-regulated protein kinase 1 and 2 (ERK1/2), c-Jun N-terminal kinase (JNK), and p38 pathways[51]. Regulation of the gene expression by MAPK can occur at transcriptional and post-transcriptional levels. Transcriptional regulation of gene expression includes activation of transcription factors and control of transcriptional initiation[52,53], while post-transcriptional regulation is via modification of mRNA processing and stability[54,55], as well as control of translational initiation[56].

In vasculatures, inhibition of ERK1/2, p38, and NF-κB activities significantly attenuates cigarette smoke extract DSP–induced up-regulation of $ET_B$ receptors, while the JNK inhibition has no such effect[34]. The anti-inflammatory medication dexamethasone strongly inhibits cigarette smoke extract DSP–induced up-regulation of $ET_B$ receptors. Thus, intracellular MAPK ERK1/2 and NF-κB inflammatory signal pathways are most likely responsible for the cigarette smoke extract DSP–induced *de novo* transcription and translation of $ET_B$ receptors[34]. Similarly, mice exposed to side-stream cigarette smoke had significantly increased airway contractile response to carbachol, ET-1, and 60 mM potassium, but airway epithelium-dependent relaxation was not affected[21]. Intraperitoneal administration of GW5074 (a Raf-1 MAPK inhibitor) or dexamethasone significantly suppressed the enhanced airway contractile responses[21]. However, exposure of rat bronchial segments to cigarette smoke extract DSP increased both $ET_A$ and $ET_B$ receptor–mediated contractions via enhanced translation of endothelin receptors[45]. Thus, targeting the MAPK-mediated up-regulation of endothelin receptors could serve as a promising novel strategy for the treatment of cardiovascular and airway diseases associated with cigarette smoke exposure.

Cigarette smoke particles are a mixture of more than 4000 different substances[57]. As a complex, it is therefore difficult to verify which substances are mainly responsible for activation of MAPK. Studies have demonstrated that polycyclic aromatic hydrocarbons (dimethylbenzanthracene and benzpyren) and oxidants, like free radicals, which are contained in cigarette smoke, induce dysfunction of arterial endothelium and SMCs[58,59]. Further studies on how cigarette smoke exposure–inducing activation of MAPK and subsequent up-regulation of endothelin receptors are still under investigations.

## PHARMACOLOGICAL TARGETS

### Endothelin Receptors

Endothelin receptor antagonists have been found to reduce blood pressure in hypertensive animals[60] and in patients with essential hypertension[61,62]. Both selective $ET_A$ and $ET_B$ receptor antagonists and nonselective endothelin receptor antagonists induce a greater vasodilatation in the forearm of hypertensive patients than in normotensive subjects[63,64]. However, an investigation on the antihypertensive efficacy and safety of darusentan, a new selective $ET_A$ receptor antagonist, demonstrated that this drug provides only a minor additional reduction in blood pressure in patients who have not attained their treatment goals with three or more antihypertensive drugs[65,66]. Major adverse effects of darusentan are related to fluid accumulation, such as edema or fluid retention, that occurred in 27% patients who received darusentan as compared with 14% for placebo[66].

In airways, both BQ-123 ($ET_A$ antagonist) and bosentan ($ET_A$/$ET_B$ receptor antagonist) prevented the development of cigarette smoke exposure–induced emphysema, blocked the expression of $ET_A$ receptors, inhibited pulmonary apoptosis, inactivated matrix metalloproteinase (MMP)-2 and MMP-9 activities in lung tissue, reduced the levels of inflammatory cytokines, TNF-α and IL-1β, and improved the biological antioxidant activity in the serum[67]. In addition, $ET_A$ receptor antagonist BQ-610 blocks cigarette smoke–induced mitogenesis in rat airways and vessels[47].

Generally speaking, targeting endothelin receptors by using their antagonists, such as bosentan, results in increased levels of ET-1 in the circulation[68]. The endothelin receptor blockers effectively antagonized both ET-1– and sarafotoxin 6c–induced responses, but they did not modify receptor up-regulation seen following organ culture[69] or following experimental cerebral ischemia[70]. There is ongoing debate about which endothelin receptor should be used clinically. Although investigation of endothelin receptor antagonists has reached as far as clinical use, new therapeutic strategies based on the underlying molecular mechanisms still need to be discovered.

## MAPK Signal Mechanisms

Theoretically, inhibition of the MAPK signal mechanisms that mediate up-regulation of endothelin receptors will exert similar therapeutic effects as endothelin antagonists and avoid excessive overflow of ET-1. In addition, inhibition of MAPK will inhibit the SMC proliferation and thus improve the adverse remodeling. A crucial role of the MAPK ERK1/2 in $ET_{B2}$ receptor up-regulation was demonstrated by Western blot in combination with specific inhibitors[34]. $ET_{B2}$ receptor up-regulation was reported for experimental focal cerebral ischemia[36] and subarachnoid hemorrhage[71], along with increased levels of $ET_B$ receptor mRNA and protein and a contractile phenotype of the receptors. The lipid-soluble smoking particles can induce activation of ERK1/2, p38, and the downstream transcriptional factor NF-κB within 3 h, with a subsequent up-regulation of vascular SMC $ET_{B2}$ receptors after 6 h of culture[34]. Inhibition of ERK1/2, p38, or NF-κB activities by their specific inhibitors significantly attenuates the lipid-soluble smoking particle–induced up-regulation of vascular SMC $ET_{B2}$[34] and $ET_A$[33] receptors. These studies on cigarette smoke exposure and MAPK-mediated mechanisms suggest that the risk factor may, via activation of MAPK-dependent NF-κB–mediated intracellular inflammatory signal transduction pathways, enhance endothelin receptor expression and subsequently result in increased contraction and proliferation of SMCs (Fig. 3). This hypothesis is supported by recent *in vivo* findings demonstrating that side-stream smoke exposure induces airway contractile hyper-responsiveness[21]. Administration of GW5074 significantly suppresses the enhanced airway contractile responses to ET-1, the smoke-induced infiltration of inflammatory cells, and mucous gland hypertrophy[21].

All three main MAPK pathways are involved in vascular[19,34] and airway[21,72,73] hyper-reactivity. After exposure to the smoke extract, it seems that ERK1/2 is more active among others in the vasculature[19,34]. However, JNK activity is predominant in airways when exposed to nicotine, the most important substance in cigarette smoke[72]. This inconsistency might be due to variations in reaction of different tissues to cigarette smoke exposure.

## CONCLUSIONS

Active and/or passive cigarette smoke exposure increase markedly the risk for developing cardiovascular and airway diseases, which are the major causes of death and disability worldwide. In this review, we have discussed recent findings on how cigarette smoke exposure induces up-regulation of endothelin receptors via activation of MAPK-mediated mechanisms in the process of developing vascular and airway hyper-reactivity, an important pathogenic characteristic of cardiovascular and airway diseases. The MAPK-mediated up-regulation of endothelin receptors provides pharmacological targets for developing novel therapeutic tools for treating cardiovascular and respiratory diseases.

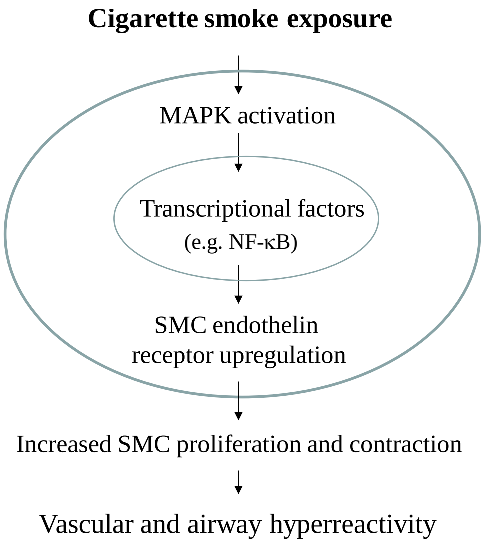

**FIGURE 3.** Schematic diagram of the MAPK-mediated up-regulation of endothelin receptors. Cigarette smoke exposure may induce the SMC endothelin receptor up-regulation via activation of MAPK-mediated NF-κB signal pathways. The endothelin receptor up-regulation may lead to increased the SMC proliferation and contraction, and subsequently cause vascular and airway hyper-reactivity. The large circle line represents the cell membrane and the small circle line indicates the nuclear membrane.

## ACKNOWLEDGMENTS

This work was supported by the Swedish Research Council (Grant No. 5958), the Swedish Heart-Lung Foundation, and the Flight Attendant Medical Research Institute (FAMRI, USA).
